# Nitric Oxide Metabolite Improves Cognitive Impairment by Reducing the Loss of Parvalbumin Inhibitory Interneurons in a Novel Mouse Model of Vascular Dementia

**DOI:** 10.2174/011570159X356939250306075324

**Published:** 2025-05-07

**Authors:** Xiaorong Zhang, Lin Cheng, Seung-Bum Yang, Moon-Se Jin, Quanyu Piao, Dae-Weung Kim, Min-Sun Kim

**Affiliations:** 1 Department of Pathology, Affiliated Hospital of Jiujiang University, Jiujiang 332000, China;; 2 Center for Nitric Oxide Metabolite, Wonkwang University, Iksan 54538, Republic of Korea;; 3 Center for Cognitive Science and Transdisciplinary Studies, Jiujiang University, Jiujiang 332000, China;; 4 Department of Neurology, Affiliated Hospital of Jiujiang University, Jiujiang 332000, China;; 5 Department of Medical Non-Commissioned Officer, Wonkwang Health Science University, Iksan 54538, Republic of Korea;; 6 Center for Nitric Oxide Metabolite, Wonkwang University Core Facility, Wonkwang University, Iksan 54538, Republic of Korea;; 7 Department of Korean Medicine, College of Korean Medicine, Wonkwang University, Iksan 54538, Republic of Korea;; 8 Department of Nuclear Medicine, Institute of Wonkwang Medical Science and Research Unit of Molecular Imaging Agent (RUMIA), Wonkwang University School of Medicine, Iksan, 54538, Republic of Korea

**Keywords:** Vascular dementia, nitric oxide, parvalbumin inhibitory interneurons, bilateral carotid artery stenosis, mouse model, stroke

## Abstract

**Background:**

This work aimed to develop a new and simple method to establish a mouse model of vascular dementia (VD). We investigated whether a new nitric oxide metabolite in the botanical mixture (a NO-donating botanical mixture, NOBM) improved learning and memory in mice that underwent bilateral carotid artery stenosis (BCAS).

**Methods:**

C57BL/6N mice received the NOBM orally (0.1 mL twice a day) after BCAS, from days 1 to 28. We assessed spatial memory using the Y maze and place recognition tests at 1 week and 4 weeks after the induction of BCAS. We quantified the parvalbumin protein in the cortex and hippocampus at 1 week and 4 weeks. We also quantified expression levels of neuronal nuclei, brain-derived neurotrophic factor, glial fibrillary acidic protein, and the number of dead neurons performed Fluoro-Jade B staining 31 days after BCAS.

**Results:**

NOBM significantly improved learning and memory behaviour in BCAS mice. Immunohistochemistry staining and Western blotting data showed a significantly higher protein expression of parvalbumin in the cortex and hippocampus of NOBM-treated BCAS animals, especially in the early stage of BCAS. Moreover, NOBM reduces neuronal loss in the cortex and reduces neuroinflammation and oxidative stress. The observed effect suggests that the NOBM reduced the loss of parvalbumin inhibitory interneurons in the early stage of VD and inhibited neuroinflammation in the VD mice model.

**Conclusion:**

Our results reveal a potential neuroprotective and therapeutic use of NOBM for cognitive dysfunction associated with cerebral hypoperfusion in a mouse model of VD.

## 1 INTRODUCTION

Vascular dementia (VD) is the second most common form of dementia after Alzheimer’s disease (AD) [1-3]. Stroke is the most direct cause of VD, with dementia developing in approximately 15-30% of subjects 3 months after a stroke [4, 5]. Cerebral hypoperfusion can initiate complex molecular and cellular inflammatory pathways and induce long-term cognitive impairment and memory loss after a stroke [6, 7]. The incidence of dementia is related to the severity and location of the stroke. After stroke, the other risk factors for VD include high blood pressure, vascular risk factors, high cholesterol, diabetes mellitus, coronary heart disease, advanced age, female sex, and low education [4, 8-10]. VD is characterized by progressive cognitive impairment, and the underlying mechanisms of VD include neuronal loss, oligodendrocyte loss, disruption of blood-brain barrier coupling, impaired clearance, astrocytosis, and microgliosis under hypoxic conditions [11-14]. In addition, glutamate excitotoxicity is another important underlying mechanism [15, 16]. In the past decade, VD research has made considerable progress; however, unlike AD, there are no effective and approved treatments.

The bilateral common carotid artery stenosis (BCAS) mouse model is an appropriate animal model to investigate VD [17, 18]. Unlike the rat model of VD, the mouse model does not involve the drawbacks of optic nerve damage [19]. Therefore, it is more suitable for behavioural experiments. At present, the BCAS model is regarded as one of the most promising VD models and is used worldwide [17, 20]. Here, we document a new sampling method to establish the BCAS mouse model using a metal wire.

Parvalbumin inhibitory interneurons (PV-INs) account for 40% of all GABAergic interneurons and form the largest class of GABAergic interneurons [21-23]. The damage or dysfunction of PV-INs contributes to the pathophysiology of several major neurodegenerative diseases, including AD, VD, and even age-related cognitive changes [23-26]. The PV-INs express Ca^2+^-binding protein parvalbumin, also called “small albumin,” which is approximately 12 kDa in humans [24]. Parvalbumin can increase the rate of Ca^2+^ sequestration, reduce Ca^2+^ levels in the neurons, protect mitochondria from Ca^2+^ overload, and prevent the activation of apoptotic pathways [27, 28]. The energy requirements of PV-INs make them highly susceptible to oxidative stress and the loss of mitochondrial membrane potential in neurodegenerative diseases [24, 29]. PV-INs can be very susceptible to hypoxia during chronic cerebral hypoperfusion. Furthermore, in two mice models of AD, the number of PV-INs in the hippocampus was found to be decreased [26, 30].

Nitric oxide (NO) is a molecule with highly diverse biological roles in physiological and pathophysiological processes [16, 31-33]. In 1998, three scientists received the Nobel Prize in Physiology and Medicine for the discovery of NO as a signaling molecule in the cardiovascular system [34]. NO can exert either protective or deleterious effects depending on its concentration and the delivered or generated location [35]. Studies show that impaired generation and signaling of NO substantially contribute to hypertension, hyperlipidemia, and neurodegenerative diseases [24, 36-38]. Lulu *et al*. [39] reported that an endothelial nitric oxide synthase (eNOS) deficit exacerbated brain white matter lesions and cognitive deficit in a BCAS mouse model. Recent studies have emphasized the involvement of the deficiency of NO in the pathophysiology of VD and focused on the potential application of a NO-related treatment for VD [40, 41]. The NO_3_^−^ -NO_2_^−^- NO pathway is a non-canonical pathway for the generation of NO in the body [42]. NO_2_^−^ and NO_3_^−^ are not simply inactive products of NO metabolism but play important roles in conserving NO. In particular, the NO_2_^−^ ion, a NO metabolite, is emerging as an endogenous signalling molecule with potential therapeutic implications for CVD (Cardiovascular disease, CVD) [43-45]. Our previous study has demonstrated that fermented garlic extract containing NO_2_^−^ ion resulted in an increase in systemic or cerebral blood flow by activating intracellular nitric oxide signalling [46, 47].

We explored the therapeutic potential of a new NOBM (the extract of fermented garlic, fermented *Scutellaria Baicalensis,* and *Rhodiola Rosea*) in a mouse model of VD based on behavioural assays, immunohistochemistry, and Western blotting.

## 2 MATERIALS AND METHODS

### 2.1 Animals

A total of 120 eight-week-old male C57BL/6N mice weighing 24-28 g were purchased from Samtako Bio Korea (South Korea). Mice were housed for at least 1 week in a controlled laboratory room (12:12 h light-dark cycle, lights on at 07:00 h, temperature: 22 ± 2°C, humidity: 45-55%) before testing. The mice had *ad libitum* access to food and water. The Laboratory Animal Ethics Committee of Wonkwang University approved all experimental procedures. All surgery was performed under anesthesia, and maximum efforts were made to minimize animals’ suffering.

### 2.2 BCAS Model and Experimental Groups

**Fig. (1):**
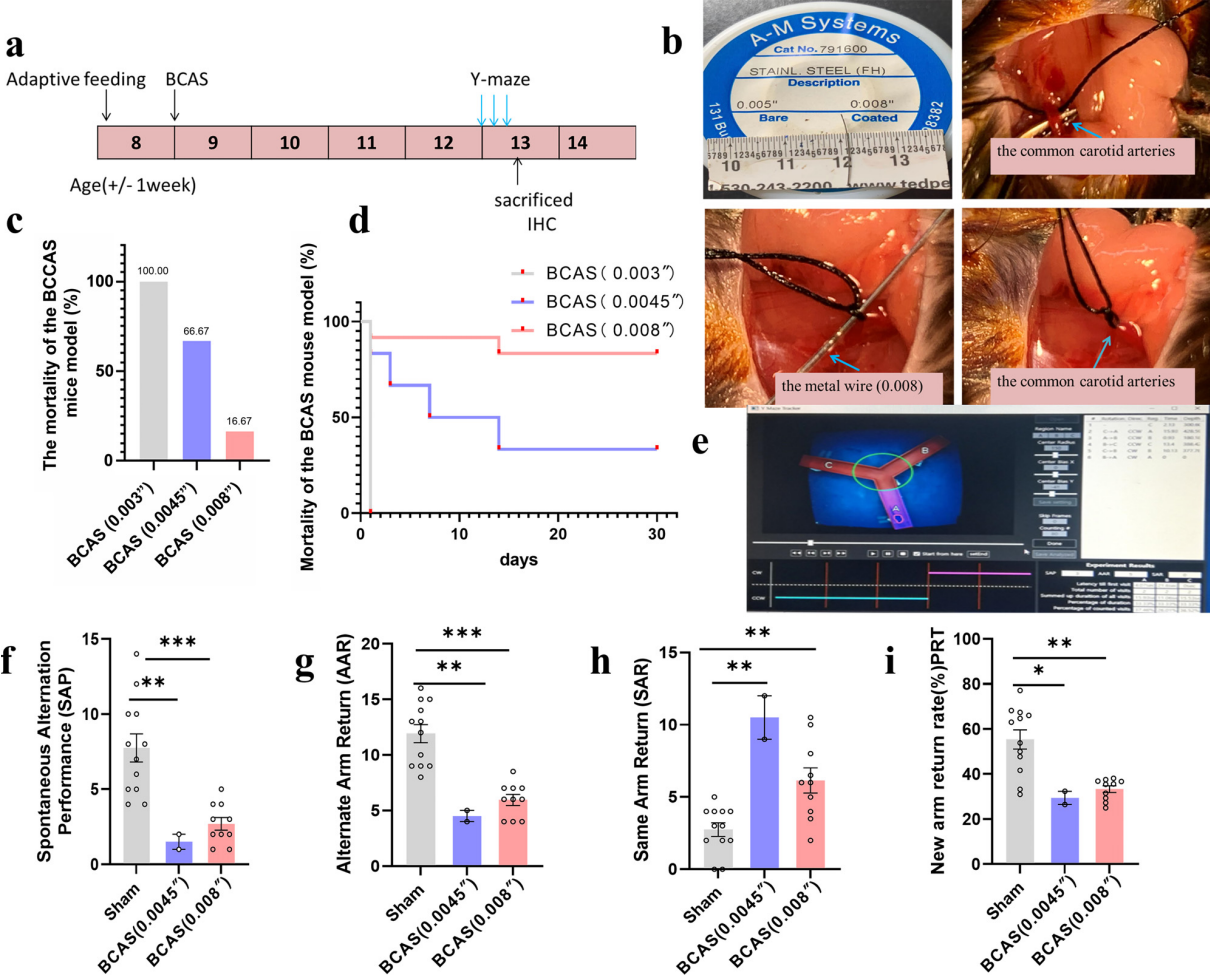
A new method was used to establish a vascular dementia (VD) mouse model. (**a**) Treatment schedule for the bilateral common carotid artery stenosis (BCAS) mouse model. (**b**) The surgical procedure of the BCAS mouse model. (**c**) Mortality of BCAS mice. (**d**) Survival curve of BCAS mice. (**e**) Y-maze analysis software screenshot. (**f**) Spontaneous alternation performance (SAP). (**g**) Alternative arm return (AAR). (**h**) Same arm return (SAR). (**i**) Place recognition test (PRT). All data are presented as the mean ± SEM. *p < 0.05, **p < 0.01, ***p < 0.001 *versus* the sham group. BCAS (0.003 inch) group, n = 6; BCAS (0.0045 inch) group, n = 6; BCAS (0.008 inch) group, n = 12.

All surgical procedures in all the groups were performed by the same researcher and accomplished within 20 min. Fig. (**1a**) shows the detailed experimental schedule. In brief, mice were anesthetized with 2% isoflurane (Baxter, USA), and the common carotid arteries (CCAs) were exposed by a midline cervical incision. The left and right CCA were exposed individually and freed from their sheaths; then, a metal wire with a diameter of 0.003, 0.0045, or 0.008 inches was twined around the artery, and a 7-0 silk suture was used to tie the CCA and the metal wire. The wire was carefully and quickly removed after ensuring that the knot was fixed (Fig. **1b**), which made partial stenosis of the artery dependent on the diameter of the metal wire. After the surgery, the mice were kept warm under observation in a heating box (28°C) until they regained consciousness and recovered enough to access food and water freely. The control mice underwent similar procedures, but their CCAs were not tied. All mice were sacrificed 31 days after BCAS for Western blotting and immunohistochemical analyses.

### 2.3 Preparation and Administration of the NOBM

The NOBM used in the experiment comprised a nitric oxide metabolite (diluted garlic extract, 2430 ppm) 1.5 mL, fermented *Scutellaria Baicalensis* 1 g, and *Rhodiola Rosea* 1g, which was diluted in 50 mL distilled water. The fermented garlic extract is a powder that is stable in the air and easy to preserve and was supplied by HumanEnos (Wanju-gun, Jeonbuk, Republic of Korea). In the 0.008″ BCAS+NO group, mice received the NOBM orally (0.1 mL, twice each day (10:00 and 16:00 hours) for 4 weeks. Meanwhile, the 0.008″ BCAS group mice only received 0.1 mL of saline orally.

### 2.4 Y-maze and Place Recognition Tests (PRT)

The Y-maze and the place recognition tests were used to evaluate spatial learning and memory and assess cognitive function and hippocampal memory deficits. The Y-maze test was performed following previously described methods 28-30 days after BCAS by an investigator blinded to the experimental groups. The angle between each arm was 120°, the length of the arm was 35 cm, the width was 10 cm, and the height was 7 cm. The movement of the mice was recorded for 5 min by a video analysis program.

The PRT was used to assess short-term memory and place recognition ability. The three arms were labeled A, B, and C. The B arm of the Y-maze was blocked, and the mice were placed in the A-arm to freely explore the C and A-arms for 10 min. After waiting for 30 min and unblocking the A-arm, the animal was allowed to explore all three arms of the Y-maze for another 10 min. The number of times the animal entered the B arm was counted to determine its spatial perception ability.

### 2.5 Immunohistochemical Assessment

Mice were deeply anesthetized with urethane (10 mg/kg) 31 days after BCAS, and perfusion fixation through the heart was performed with chilled 1% paraformaldehyde (PFA), followed by 4% PFA. Once the perfusion was completed, the brain was harvested and fixed in a 4% PFA solution overnight at 4°C. Mice brains were then immersed in a 30% sucrose solution diluted with PBS for 1-2 days at 4°C. We prepared 35-µm thick tissue sections using a freezing microtome (Leica, Germany) and stored them in a tissue storage solution at −20°C. Brain sections were then washed with phosphate-buffered saline (PBS) and incubated for 4 min with antigen retrieval at 95°C. Sections were rinsed for 10 min with PBS and incubated with 0.5% PBS-T containing 3% goat serum for 10 min. Then, the sections were incubated overnight at 4°C with primary antibodies for parvalbumin (1:2000, Abcam, #ab11427, USA) and NeuN (1:1000, Abcam, #ab128886, USA). Next, sections were washed with 0.05% PBS-T three times and incubated for 1 h at room temperature with the secondary antibody (Polymer HRP anti-Rabbit IgG; GBI Labs, Cat No.: D13-110, USA). The sections were then washed with 0.05% PBS-T at 38°C, rinsed with an antigen retrieval solution for 5 min at room temperature, and visualized with 0.05% diaminobenzidine (DAB) hydrochloric acid and 0.003% H_2_O_2_. Sections were rinsed with 0.1 M PB, mounted on gel-coated slides, air-dried, and dehydrated. Finally, the sections were placed on coverslips and observed under a light microscope. Three sections per brain and three fields per section were used for quantification, and the ImageJ software (1.8.0) was used to calculate the total number or strength of positive cells in each field.

### 2.6 Western Blot Analysis

The cortex and hippocampal samples (n = 4 per experimental group) were homogenized with cold RIPA lysis buffer (Thermo Scientific, USA) and protease and phosphatase inhibitor cocktails (Thermo Scientific, USA), followed by centrifugation at 13,000 rpm for 20 min. Protein concentration was measured using a BCA Protein Assay Kit. Protein samples (20 μg) were separated into 10%, 12%, or 15% SDS-PAGE gels and transferred onto Immobilon P membranes. Blocking was performed by incubating the membranes with 5% non-fat dry milk that dissolved with the 1× TBST (Tris-buffered saline containing 0.1% Tween 20), pH 7.4. The membranes were incubated overnight at 4°C under continuous agitation with the primary antibody for GFAP (1:5,000, Abcam, #ab7260, USA), parvalbumin (1:1,000, Abcam, #ab11427, USA), BDNF (1:1,000, Abcam, #ab108319, USA), or β-actin (1:1,000, Calbiochem, #D00024369, USA) diluted in TBS-T. The membranes were washed with TBS-T three times for 10 min and incubated with HRP-linked anti-rabbit IgG (1:1,000 in TBS-T, Santa Cruz Biotechnology #sc-2357, USA) or anti-mice IgG (1:1,000 in TBS-T, Cell Signaling #7076S, USA) for 1 h at room temperature. Then, the membranes were washed three times with TBS-T, and the immune-reactive bands were revealed using an enhanced chemiluminescent substrate (Thermo, #34577, USA). Protein levels were quantified with ImageJ software, and the ratios of GFAP, BDNF, and PV/β-actin were calculated.

### 2.7 Fluoro-Jade B Staining

The Fluoro-Jade B (FJB; Histo-Chem Inc #US 6229024 B1, USA) staining procedure was performed exactly as previously described [41]. Frozen sections (25 µm) were rinsed in distilled water for 10 min to remove the storage solution and incubated in an 80% alcohol solution containing 1% sodium hydroxide for 5 min. The sections were transferred to a solution of 0.06% potassium permanganate for 10 min, followed by 70% alcohol for 2 min and distilled water for 2 min. The sections were then rinsed in distilled water for 2 min and exposed to a 0.01% working solution of FJB at room temperature for 15 min. Finally, sections were mounted with distilled water onto gelatine-coated slides, followed by coverslip mounting. The brain slide was examined by fluorescence microscopy. For the quantification of FJB+ neurons, two researchers calculated the number of FJB^+^ neurons in each randomized microscopic field of PFC (200X) independently from each mouse by using ImageJ, and the results were the average values of numbers of each mouse.

### 2.8 Statistical Analysis

One-way analysis of variance (ANOVA) was used for comparison using GraphPad Prism 8.0 (GraphPad Software, La Jolla, CA, USA). The statistical analysis values were expressed as the mean ± SEM. Mortality was presented as Kaplan-Meier survival graphs. The Y-maze results were analyzed with the Kruskal-Wallis test. A *p*-value of < 0.05 was considered statistically significant.

## 3 RESULTS

### 3.1 The New Method Successfully Established a BCAS Mouse Model

First, 8-week-old C57BL/6N mice underwent adaptive feeding for 1 week. Then, the VD model was generated using a metal wire of different diameters. After 4 weeks, the mice were tested for cognitive deficits using the Y-maze test. The mice were then sacrificed for immunohistochemistry analysis. We aimed to identify an appropriate diameter of the wire for a satisfactory degree of stenosis, which would imitate VD. Fortunately, we successfully identified a suitable wire to establish a BCAS mouse model using this new method.

#### 3.1.1 Mortality Rate of BCAS Mice with the Different Metal Wires

We observed the mortality rate of mice within 30 postoperative days and sacrificed the mice on day 31. We found that the mortality ratio was 100% (6/6) in the BCAS (0.003 inch) group, and all mice died within 24 hours after surgery. By contrast, 66.67% (4/6) died in the BCAS (0.0045 inch) group within 30 postoperative days, and the times of animal death were on postoperative days 1, 3, 7, and 14. In the BCAS (0.008 inch) group, the mortality was 16.67% (10/12) (Fig. **1c**). The two mice died on postoperative days 1 and 14. The Kaplan-Meier survival curves and the log-rank (Mantel-Cox) test showed better overall survival in the BCAS (0.008 inch) group than in the BCAS (0.0045 inch) group (Fig. **1d**).

#### 3.1.2 Cognitive Deficits in the BCAS (0.008 Inch) Model

To confirm whether this new BCAS model method (0.008 inch) induced cognitive deficits, the Y-maze test was employed (Fig. **1e**). Continuous spontaneous alternation is a behavioural test used to measure working memory in the Y-maze test [48]. Mice typically prefer to investigate a new arm of the maze rather than return to a previously visited one. Using the natural exploratory behaviour of mice, the spontaneous alternation Y-maze test can assess short-term spatial working memory. In this part, we counted the spontaneous alternation performances (SAP), the alternative arm return (AAR), the same arm return (SAR), and the percentage of new arm visiting rate in the place recognition test (PRT) 4 weeks after the operation.

We found that the average SAP and AAR of the BCAS (0.008 inch) surgery group was significantly lower than that of the sham-mice group (*p <* 0.05) (Fig. **1f**, **g**). The SAR of the BCAS (0.008 inch) group was higher than that of the sham-mice group (*p <* 0.05) (Fig. **1h**). Taken together, these data indicate that the BCAS (0.008 inch) group had impaired hippocampus-dependent spatial memory. The PRT experiment revealed that the new arm visit rate was twice lower in BCAS (0.008 inch) mice than in sham mice. Short-term memory was impaired significantly in the BCAS (0.008 inch) group (Fig. **1i**). These results indicate that the BCAS (0.008 inch) operation significantly impaired hippocampus-dependent spatial memory and short-term memory. This indicated that the model was successful. The BCAS (0.045 inch) group also showed significant changes in these data, but there were only two mice in this group.

#### 3.1.3 Neuronal Loss in the BCAS Mouse Model

**Fig. (2):**
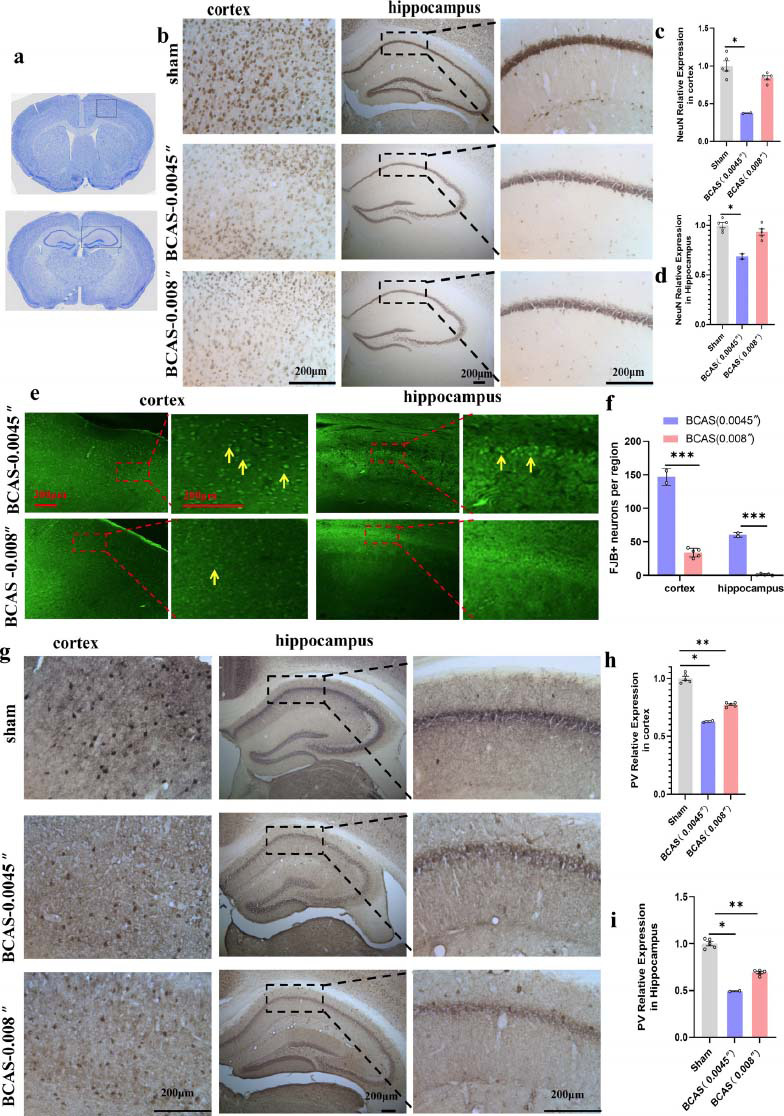
Neuron loss in the bilateral common carotid artery stenosis (BCAS) model. (**a**) The atlases used for selecting the evaluated tissue sections (the area inside the black dotted box). (**b**) NeuN immunostaining in the cortex (original magnification, 400×) and hippocampus (original magnification, 100×). (**c**, **d**) Quantitative analysis of NeuN expression in the cortex (**c**) and hippocampus (**d**). (**e**, **f**) Fluoro-Jade B fluorescence staining in the cortex and hippocampus, the FJB positive neurons are marked by yellow arrows. (**g**) Parvalbumin immunostaining in the cortex (original magnification, 400×) and hippocampus (original magnification, 100×) (**h**, **i**) Quantitative analysis of parvalbumin expression in the cortex (**h**) and hippocampus (**i**). All data are presented as the mean ± SEM. *p < 0.05, **p < 0.01 *versus* the sham group, ***p < 0.001 *versus* BCAS 0.008 inch group. BCAS (0.0045 inch group, n = 2; BCAS 0.008 inch, n = 5; sham group, n = 5).

The results indicated that the number of total neurons marked by neuronal nuclei (NeuN, a key RNA-associated protein expressed in all neurons) was significantly decreased in the cortex (Figs. **2b**, **c**) and hippocampus (Figs. **2b**, **d**) of the BCAS (0.0045 inch) group, and were prominently decreased in the III-IV layer of the cerebral cortex. In the BCAS (0.008 inch) group, the expression of NeuN was slightly reduced, but there was no significant difference compared with the sham group (Figs. **2c**, **d**).

FJB staining was used to identify the degenerating neurons [49]. In the BCAS (0.0045 inch) group, there were several FJB-positive neurons in the cortex and hippocampus. In the BCAS (0.008 inch) group, there were some FJB-positive neurons in the cortex but not in the hippocampus (Figs. **2e**, **f**). The number of FJB-positive neurons was higher in the cortex of the BCAS (0.008 inch) group than in the sham group. Interestingly, the expression of NeuN in the BCAS (0.008 inch) group was not significantly different in the cortex and hippocampus compared with the sham group, but the FJB-positive neurons were increased in the cortex.

Parvalbumin is a calcium-binding protein that contributes to maintaining the excitatory/inhibitory imbalance. A reduced parvalbumin expression induces excitotoxicity. The expression of parvalbumin in the cortex and hippocampus of the BCAS (0.0045 inch) group was significantly lower than in the BCAS (0.008 inch) and sham groups (Figs. **2g**-**i**). These results suggest that BCAS induced a downregulation in parvalbumin expression, with a significant difference between the BCAS (0.0045 inch) and BCAS (0.008 inch) groups (*p <* 0.05) (Figs. **2h**, **i**). The loss of PV-INs in the cortex may explain the increase of FJB-positive cells, but no significant difference was noted in the number of NeuN. Another likely reason is that NeuN cannot be used to distinguish between living and degenerating cells.

### 3.2 NOBM Function in the BCAS (0.008 Inch) Model

#### 3.2.1 NOBM Alleviated Cognitive Impairment after BCAS

**Fig. (3):**
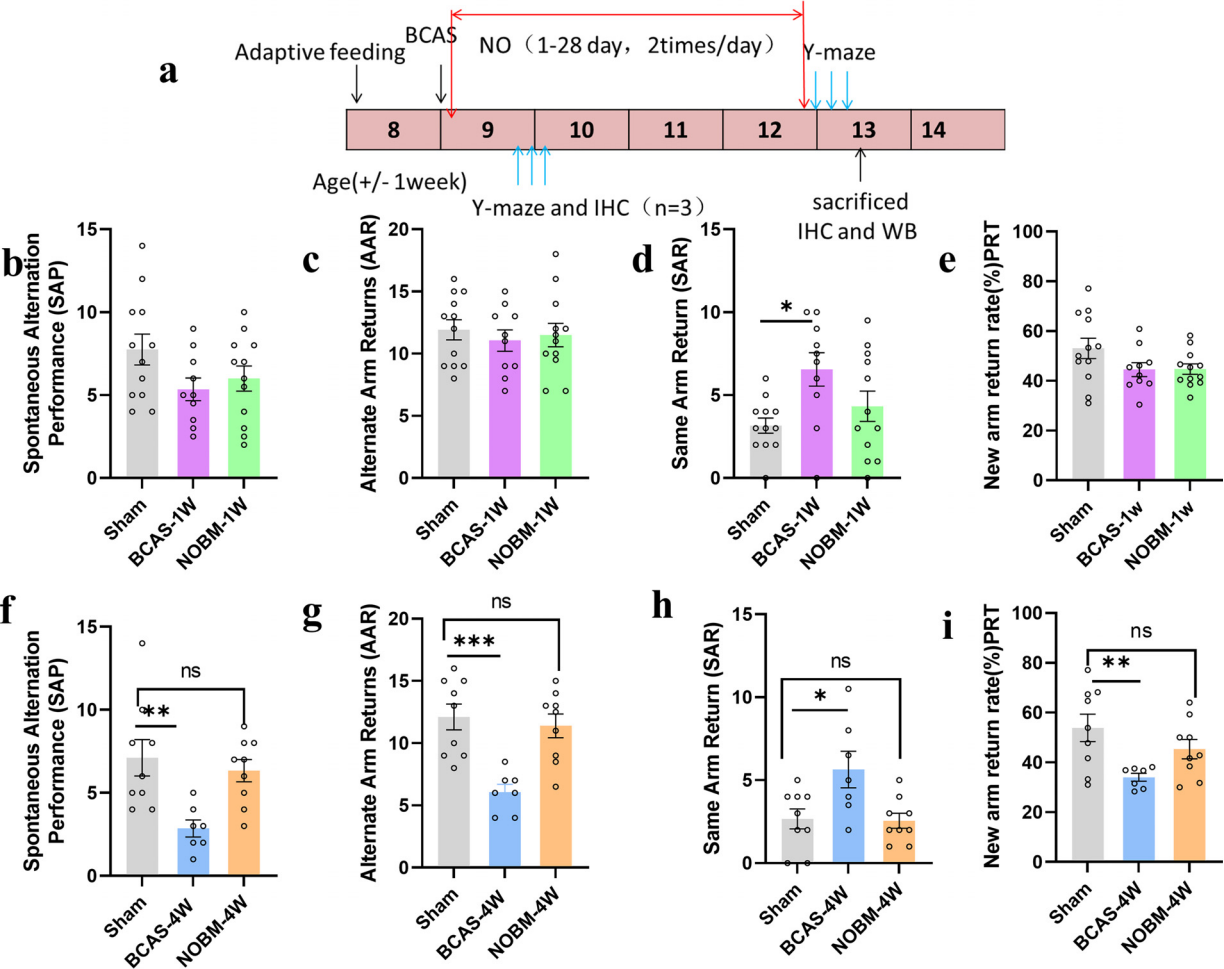
The NO-donating botanical mixture (NOBM) alleviated cognitive impairment after bilateral common carotid artery stenosis (BCAS). (**a**) Treatment schedule for the BCAS mouse model. (**b**) Number of spontaneous alternation performances (SAPs) at 1 week. (**c**) Alternative arm return (AAR) at 1 week. (**d**) Same arm return (SAR) at 1 week. (**e**) Percentage of new B arm visiting rate in the place recognition test (PRT) at 1 week. (**f-i**) SAP (**f**), AAR (**g**), SAR (**h**), and PRT (**i**) at 4 weeks. All data are presented as the mean ± SEM. *p < 0.05, **p < 0.01, ***p < 0.001 *versus* sham group. n = 10-12 per group at 1 week, n = 7-9 per group at 4 weeks.

After successfully establishing the BCAS (0.008 inch) mouse VD model, we assessed the effect of a NOBM on VD. The flowchart of the study is shown in Fig. (**3a**). We used the Y-maze test to evaluate the short-term working memory of mice at 1 week and 4 weeks. At 1 week, the BCAS and sham groups had similar SAP (Fig. **3b**), AAR (Fig. **3c**), and PRT (Fig. **3e**) results. The SAR (Fig. **3d**) was increased in the BCAS group. This suggested that there was no significant cognitive impairment at 1 week after BCAS. At 4 weeks, the sham group exhibited a high rate of spontaneous alternations, whereas this rate was lower in the BCAS group. However, the rate of spontaneous alternations (Fig. **3f**) and AAR (Fig. **3g**) were significantly increased in mice that had received NOBM. The SAR was increased in the BCAS group, but it could be reduced by the NOBM (Fig. **3h**). In the PRT, the sham group exhibited a high rate of new arm visits; however, BCAS induction reduced this rate, and the NOBM restored it (Fig. **3i**). Thus, NOBM administration led to a better outcome in the Y-maze test, which is used to measure short-term spatial working memory.

#### 3.2.2 Effect of the NOBM on the Number of Neurons in BCAS Animals

**Fig. (4):**
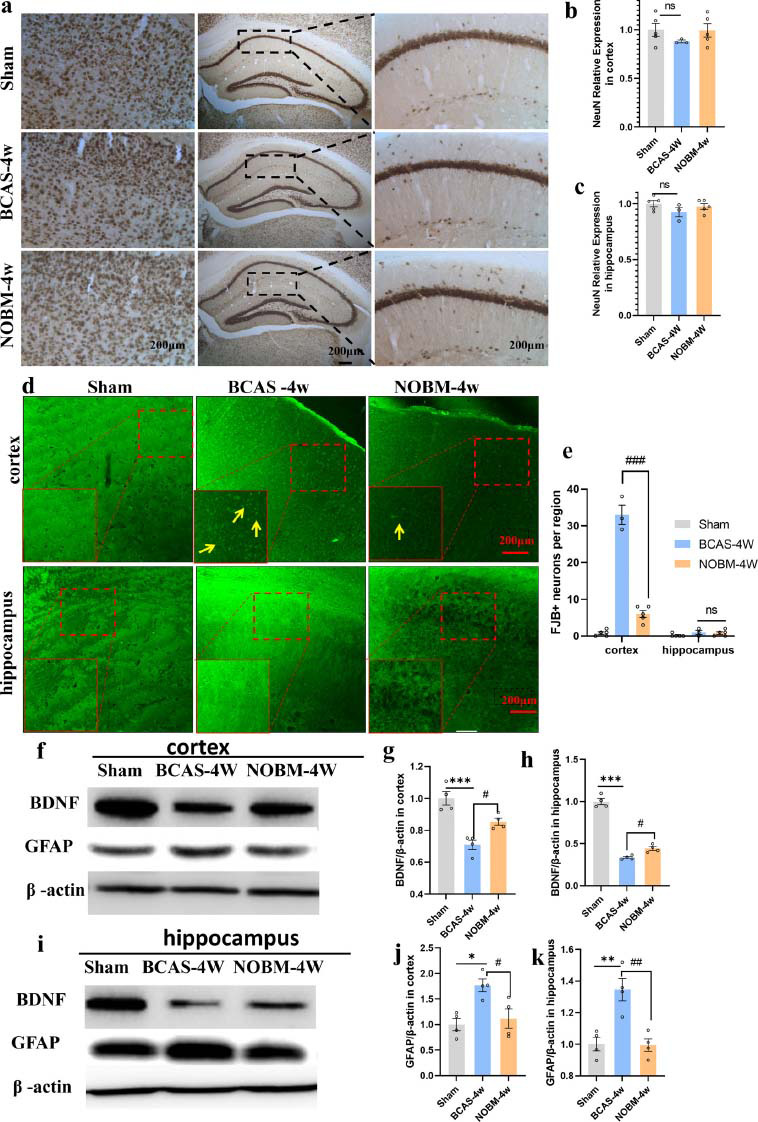
The NO-donating botanical mixture (NOBM) alleviated neuronal loss and neuroinflammation and upregulated BDNF expression after bilateral common carotid artery stenosis (BCAS). (**a**) NeuN immunostaining in the cortex and hippocampus. (**b**, **c**) Quantification of NeuN in the cortex and hippocampus. (**d**, **e**) Fluoro-Jade B fluorescence staining in the cortex and hippocampus, the FJB positive neurons are marked by yellow arrows. (**f**) BDNF and GFAP expression in the cortex was assessed by Western blotting. (**g**, **h**) Quantification of proteins in the cortex. (**i**) BDNF and GFAP expression in the hippocampus assessed by Western blotting. (**j**, **k**) Quantification of proteins in the hippocampus. #*p* < 0.05, ##*p* < 0.01, ###*p* < 0.001 *versus* BCAS mice; *p < 0.05, **p < 0.01, ***p < 0.001*versus* sham group, n = 3-5 per group.

Immunohistochemical staining showed no significant change in the levels of NeuN-positive neurons in the cortex and hippocampus between the BCAS model and sham group (Figs. **4a**-**c**). However, consistent with previous results, many FJB-positive cells appeared in the cortex of the BCAS mice. FJB-positive cells were barely observed in the cortex of mice treated with NOBM and the sham group. There was no significant neuronal loss in the hippocampus in any of the study groups (Figs. **4d**, **e**). This result indicated that NO treatment could alleviate neurodegenerative processes in VD. The loss of NeuN-positive neurons was not significant, but there were some degenerated neurons in the cortex. These degenerated neurons were mainly parvalbumin-positive interneurons.

Next, we quantified BDNF expression by Western blotting in the cortex and hippocampus. BCAS mice had significantly lower BDNF levels in the cortex (Figs. **4f**, **g**) and hippocampus (Figs. **4h**, **i**) than sham mice; NOBM alleviated or reversed this reduction (*p <* 0.05).

Glial fibrillary acidic protein (GFAP) is an astrocyte marker that can reflect the intensity of neuroinflammation to a certain extent. GFAP expression was significantly increased in the cortex (Figs. **4f**, **j**) and hippocampus of BCAS mice compared to that of sham mice (Figs. **4i**, **k**); NOBM alleviated this effect (cortex; *p <* 0.05, hippocampus; *p <* 0.01).

#### 3.2.3 NO Inhibited the Loss of Parvalbumin Neurons in the Early Stage of VD

**Fig. (5):**
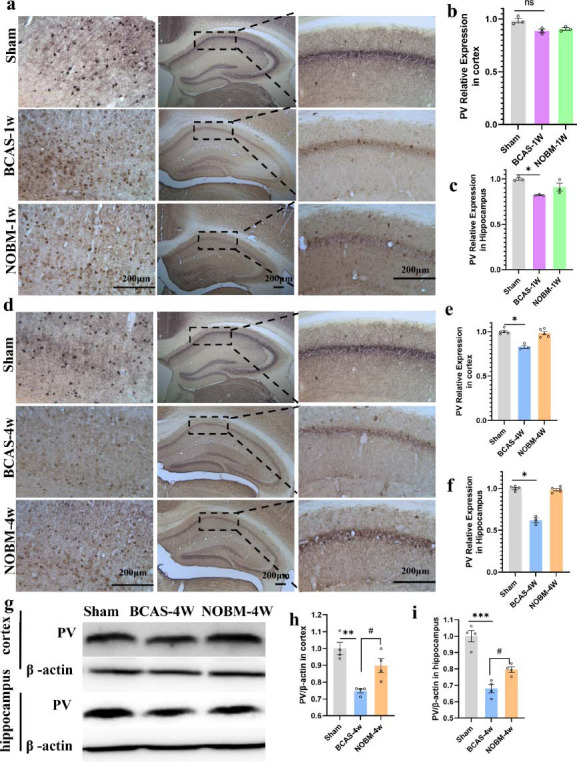
The NO-donating botanical mixture (NOBM) alleviated the loss of parvalbumin inhibitory interneurons (PV-Ins) in the early stage of ischemia. (**a**) Parvalbumin immunostaining in the cortex and hippocampus at 1 week. (**b**, **c**) Quantification of parvalbumin in the cortex and hippocampus at 1 week. (**d**) Parvalbumin immunostaining in the cortex and hippocampus at 4 weeks. (**e**, **f**) Quantification of parvalbumin in the cortex and hippocampus at 4 weeks. (**g**) Parvalbumin expression in the cortex assessed by Western blotting. (**h**, **i**) Quantification of proteins in the cortex and hippocampus. #*p <* 0.05 *versus* BCAS mice; *p < 0.05, **p < 0.01, ***p < 0.001 *versus* sham group, n = 3-5 per group.

One week after the surgery, the BCAS and sham groups had comparable parvalbumin-positive neuron populations (Figs. **5a**-**c**). However, in the hippocampus, especially in the CA1 area, BCAS significantly reduced the number of parvalbumin-positive neurons (Fig. **5c**, *p <* 0.05). Interestingly, in the hippocampus, the expression of parvalbumin-positive neurons was not significantly different in the BCAS NOBM group compared with the sham group. This suggested that the loss of parvalbumin-positive neurons occurred in the early stage of ischemia, and NOBM treatment could reverse the reduction.

At 4 weeks, the expression of parvalbumin in the BCAS group was significantly reduced in the cortex and the hippocampus compared with the sham group (Figs. **5d**-**f**); however, the NOBM could reverse this decline. This suggests that NO inhibited the loss of the parvalbumin-positive neurons. These experiments revealed that the hippocampus was more sensitive to ischemia than the cortex in this model, and the NOBM could inhibit the loss of parvalbumin-positive neurons early in the hippocampus. Furthermore, the Western blotting results confirmed that NO inhibited the loss of parvalbumin protein (Fig. **5g**-**i**). The expression of parvalbumin in the BCAS group was significantly decreased in the cortex (Fig. **5g**, **h**) and hippocampus compared with the sham group (Fig. **5g**, **i**), where it was upregulated by the NOBM (cortex; *p <* 0.05, hippocampus; *p <* 0.05).

## 4 DISCUSSION

BCAS mouse models are well-characterized and have been increasingly used to study vascular cognitive impairment, as they do not involve optic nerve damage and are considered more suitable than rat models for behavioural experiments [50]. Neuron damage, inflammation [51], astrogliosis [52], microgliosis [53], oxidative stress, white matter damage [51, 54] and cognitive dysfunction [55-60] are the pathological hallmarks of VD. We demonstrated that the 0.008-inch metal wire could be used to successfully model VD, causing obvious memory impairment observable in the Y-maze and PRT tests. In addition, the surgical process used in our study to establish the VD mouse model was easier and more cost-effective than the existing steel micro-coil technology [17].

**Fig. (6):**
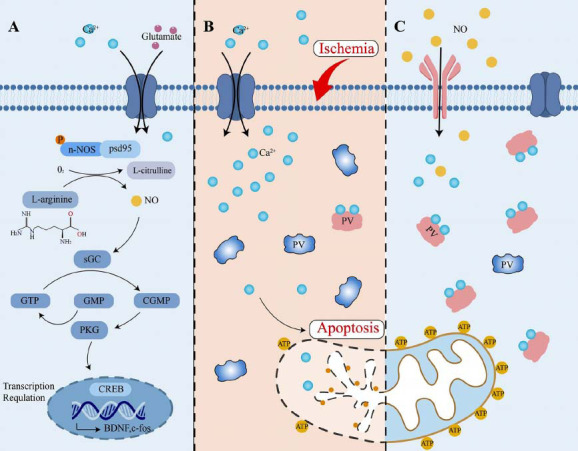
The NO-sGC-cGMP-PKG pathway and the function of the parvalbumin protein. (**A**) Ca^2+^ influx through NMDAR on the postsynaptic membrane activates nNOS by binding with PSD95, leading to NO production. NO activates soluble guanylate cyclase (sGC), producing cGMP, which interacts with PKG. PKG leads to CREB phosphorylation, leading to the transcriptional activation of various genes, such as BDNF and c-fos. (**B**) Under ischemic conditions, a large Ca^2+^ influx and NO deficiency induce the inactivation of parvalbumin, resulting in a Ca^2+^ overload in mitochondria and inducing apoptosis. (**C**) Exogenous supplementation of NO can activate a large amount of parvalbumin, which can bind to Ca^2+^ and keep the mitochondria active [57-60].

The present study also shows exogenous treatment with the NO_2_^-^ ion, an NO metabolite in the herbal mixture, can reduce neuron damage, decrease astrocyte activation and attenuate cognitive deficits in VD mice. NO upregulated parvalbumin expression levels, reducing the loss of PV-INs in the early stage of VD. During the early stages of ischemia and hypoxia, the increase in NMDA receptors aggravates the accumulation of glutamate, which continues to stimulate NMDA receptors and generates an excessive Ca^2+^ influx [61]. Ultimately, calcium overload in the cytoplasm and mitochondria of the neurons results in mitochondrial damage, neuronal injury, and cognitive dysfunction [62, 63]. Parvalbumin is a small 12 kDa protein molecule that is expressed in PV-INs. Parvalbumin can increase the rate of Ca^2+^ sequestration, protect mitochondria from Ca^2+^ overload, and prevent the activation of apoptotic pathways [64]. Therefore, parvalbumin can alleviate the excitotoxic effect of glutamate and reduce neuronal loss. Here, we found that the NO metabolite significantly reduced the loss of PV-INs in the early stages of VD (around 1 week), resulting in mitochondrial protection from Ca^2+^ overload. The NO metabolite likely improves cognitive impairment in VD through this mechanism (Fig. **6**). Another study suggested that PV-INs in the deep layers of frontal cortical regions were especially vulnerable in AD mice [65]. Researchers demonstrated that ameliorating parvalbumin-positive neuronal deficits significantly ameliorated memory deficits and reversed AD pathological processes in App^NL-G-F/NL-G-F^ AD mice [66]. This evidence suggests that the density of PV-INs was closely related to cognitive impairment, and our study demonstrated the importance of PV-INs in VD. Neuronal loss is an important pathological manifestation of VD. Our results show that the loss of neurons in VD is most significant in terms of loss of parvalbumin neurons, and the NO metabolite can significantly reduce the loss of parvalbumin neurons and improve cognition deficits [67-69].

In our study, the Y-maze test demonstrated that the NO metabolite significantly improved spatial memory and cognitive impairment in BCAS mice. Furthermore, BDNF, a neurotrophic factor contributing to neuronal survival, was upregulated in the NOBM treatment group. A previous study also showed that enhanced eNOS phosphorylation or increased NO content regulates neurogenesis [70]. Additionally, in our previous study, oral treatment of fermented garlic extract with the nitrite ion used in this study caused activation of the eNOS system in the systemic vessels, upregulation of regional cerebral blood flood, and protection of ischemic injury in the heart [46, 47, 71-76].

In the present study, the expression of GFAP (a maker of astrocytes) was significantly decreased in the NOBM group compared with the control group. This suggests that NO can inhibit the activation of astrocytes and reduce neuroinflammation in VD.

Currently, there is no specific treatment strategy for VD [3], but NO has therapeutic potential. Our study confirmed that NO metabolite treatment could decrease neuronal loss, inhibit inflammation, and improve cognitive impairment in mice with VD. Furthermore, we found that the NO metabolite improved cognitive impairment by reducing the loss of parvalbumin-positive neurons. These results indicate that the NO metabolite is a therapeutic agent for the prevention and treatment of VD.

Our study has some limitations. First, although NO ameliorated cognitive impairment in the VD mouse model by reducing the loss of parvalbumin neurons, the specific underlying regulatory mechanism requires further exploration. In the future, we plan to study specifically related pathways such as ferroptosis, programmed necrosis, and apoptosis. Second, we did not assess the effect of NO metabolite only in parvalbumin-positive neurons *in vivo*, nor the level of glutamate in the BCAS mouse model. In the future, more studies can be carried out using electron microscopy and neuro-electrophysiological technology to explore the specific mechanism by which NO inhibits the loss of PV-INs in VD.

## CONCLUSION

To our knowledge, this is the first report of using a 0.008-inch metal wire technique to successfully establish a VD mouse model. Our results showed that an NOBM could increase BDNF expression, regulate astrocyte counts and improve cognitive impairment in VD mice. More importantly, a NOBM can reduce the loss of parvalbumin neurons; this may be why NO metabolites in the herbal mixture result in improved cognitive impairment in VD mice. Thus, NO metabolites in the herbal mixture have therapeutic potential in the treatment of VD. Additional experimental and clinical studies will be required to assess the potential clinical applications of NO metabolites in VD.

## Data Availability

Data is available on request from the authors. The data that support the findings of this study are available from the corresponding author upon reasonable request. Some data may not be made available because of privacy or ethical restrictions.

## LIST OF ABBREVIATIONS

AAR: Alternative Arm Return
AD: Alzheimer’s Disease
BCAS: Bilateral Common Carotid Artery Stenosis
eNOS: Endothelial Nitric Oxide Synthase
GFAP: Glial Fibrillary Acidic Protein
NO: Nitric Oxide
PRT: Place Recognition Test
PV-INs: Parvalbumin Inhibitory Interneurons
SAP: Spontaneous Alternation Performances
VD: Vascular Dementia
